# Epigenetic regulation of general anesthesia-induced neonatal neurodegeneration

**DOI:** 10.18632/oncotarget.14568

**Published:** 2017-01-10

**Authors:** Min Jia, Mu-Huo Ji, Jian-Jun Yang

**Affiliations:** ^1^ Department of Anesthesiology, Zhongda Hospital, Medical School, Southeast University, Nanjing, China

**Keywords:** histone acetylation, HDAC3, hippocampus, neonate, general anesthesia

General anesthesia-induced neurocognitive dysfunction in the developing brain has been well documented [1], yet the underlying mechanisms remain incompletely understood. Epigenetic mechanism is thought to be a key regulator of gene-environment interactions and have been implicated in the etiology of various brain disorders. Histone acetylation is one of the most studied epigenetic modifications and controlled by the opposing activities of histone acetyltransferases (HATs) and histone deacetylases (HDACs), which add or remove acetyl groups from protein substrates, respectively. Although its regulation is extraordinarily complex and dynamic, it is generally considered that an increase in histone acetylation induces chromatin structure from a tightly packed configuration to a loose one and subsequently results in transcriptional activation. By contrast, a decrease in histone acetylation mediated by HDACs condenses chromatin structure and leads to transcriptional silencing. There is accumulating evidence indicating that aberrant histone acetylation plays a critical role in the cognitive impairment that is associated with psychiatric and neurodegenerative disorders. Presently unclear, however, is how altered histone acetylation participates in the pathogenesis of general anesthesia-induced neonatal neurocognitive impairments.

Our recently published study in Neurobiology of Disease reported that neonatal exposure to sevoflurane induces neurobehavioral impairments with concurrent long-lasting down-regulation of hippocampal histone acetylation. To further address the causal role of histone acetylation, we normalized histone acetylation by using class I HDACs inhibitor sodium butyrate (NaB) and found the improved neurobehavioral abnormities [2], indicating that histone acetylation plays a key role in sevoflurane exposure induced-neurobehavioral impairments. Of note, our study further showed that normalization of hippocampal histone acetylation after NaB treatment could rescue the decreased levels of synapse-related proteins, including BDNF, synapsin1, PSD95, pCREB/ CREB, CBP, and restore the loss of dendritic spines in the CA1 pyramidal neurons. Although our study did not exclude that additional mechanisms are involved, our results suggested a potential mechanism by which down-regulated hippocampal histone acetylation can participate in general anesthesia-induced cognitive impairments in neonates.

The highly expressed class I HDACs (including HDAC1, 2, 3 and 8) in the central nervous system suggest broad roles of these enzymes in controlling histone acetylation and transcription. Taking into account that NaB belongs to a pan-HDACs inhibitor, the question of contribution of individual HDACs toward general anesthesia-induced neonatal neurocognitive abnormalities arises. Using a combination of pharmacological and genetic approaches, it has been suggested that HDAC2 but not HDAC1 is a specific negative regulator of synaptic plasticity and memory formation [3]. Unlike HDAC1 and HDAC2, HDAC3 appears to be uniquely recruited to the silencing mediator for retinoic acid and thyroid hormone receptor (SMRT) or nuclear receptor corepressor (NCoR) complex *via* inositol tetraphosphate [4], and the activation of HDAC3 is mediated by a deacetylase activating domain (DAD) presented in the SMRT or NCoR [5]. It has been revealed that HDAC3 possesses selective toxicity in neurons and appears to be a negative regulator of spatial memory formation and extinction of addiction-like behaviors [6]. Although genetically modified mice are needed to understand the specific roles of HDACs in general anesthesia-induced neonatal neurotoxity in our future studies, we have already found that HDAC3, but not HDAC2, was up-regulated in the CA1 of hippocampus in neonates after repeated sevoflurane exposures [2], which suggested that HDAC3 might be specifically involved in sevoflurane anesthesia-induced neonatal neurocognitive dysfunction.

Moreover, HDAC3 can directly interact with most of the class II HDACs proteins, particularly HDAC4, 5, 7, and 10, which is important for its molecular and neurobehavioral consequences [7]. In addition, it has been shown that HDAC3 can be recruited to some active gene promoters, such as c-fos and Nr4a2, *via* methyl CpG binding protein 2 (MeCP2) (binds specifically to methylated DNA and turns off the gene transcription) and positively regulates a subset of neuronal genes *via* forkhead box o (FOXO) deacetylation, leading to cognitive and social impairments in animal model of Rett syndrome [8]. Besides, it is now recognized that HDAC3 can also regulate non-histone proteins, such as myocyte enhancer factor 2 (MEF2), brain derived neurotrophic factor (BDNF) and nuclear factor-kappaB (NF-κB) protein subunit-RelA, all of which are required for the gene regulation of structural plasticity and gene induction of long-term memory formation. These mechanisms thus state compelling evidence to develop specific HDAC3 inhibitor as therapeutic for treating general anesthesia-induced neonatal degeneration in the developing brain. Our study delineates an epigenetic mechanism that underlies general anesthesia-induced neonatal neurocognitive abnormalities, providing a potential drug target (HDAC3), for the treatment of general anesthesia-induced neonatal neurotoxity. However, one key issue is to identify the specific HDAC in general anesthesia-induced neonatal neurocognitive abnormalities, thus develop class- and isoform-specific HDAC inhibitors to minimize the unintentional side effects. Clearly, despite recent advancements in the comprehension of the role of HDAC3 in the regulation of long-term memory formation, many questions on other potential functions of HDAC3 remain open.
